# Higher free-roaming dog density sustains rabies virus transmission in Haiti

**DOI:** 10.1038/s41598-026-35359-y

**Published:** 2026-01-24

**Authors:** Andrew J. Beron, Ravikiran Keshavamurthy, Cassandra Boutelle, Ryan Wallace

**Affiliations:** 1 Centers for Disease Control and Prevention, Atlanta, GA USA; 2 Epidemic Intelligence Service, Centers for Disease Control and Prevention, Atlanta, GA USA

**Keywords:** Diseases, Microbiology

## Abstract

Eliminating dog-to-dog rabies virus transmission, the primary cause of > 70,000 human deaths annually, remains a challenge in over 100 countries due to the difficulty of implementing effective dog vaccination and population management programs. Despite the development of tools to optimize vaccine impact, rabies virus transmission dynamics are still not well-understood, largely due to insufficient surveillance. Utilizing data from Haiti’s advanced rabies surveillance system, we analyzed likely rabies cases, adjusted for a 5% detection rate, to estimate the true rabies burden in Haitian dogs. Our study calculated the effective reproduction number (Re) of rabies, finding strong associations between Re and free-roaming dog density, with Re falling below 1.0 when free-roaming dog density fell below 10 per km². This association suggests that denser free-roaming dog populations may perpetuate rabies transmission, providing critical insights for targeting effective vaccination efforts.

**Supplementary Information:**

The online version contains supplementary material available at 10.1038/s41598-026-35359-y.

## Introduction

Dog-maintained rabies viruses are present in over 100 countries and spillover into humans through bites and scratches results in over 70,000 deaths annually^1,2^. Vaccines for both people and dogs were developed over 130 years ago, yet access to and appropriate utilization of these interventions have not been fully embraced in many low- and middle-income communities^3^. Sustained herd immunity of 70% of at-risk dogs has been shown mathematically and supported empirically to halt transmission, and if maintained long enough, will eventually eliminate the virus from the population^4^. The definition of at-risk dogs is often debated, but per the origin of the mathematically derived 70% herd immunity goal, this is defined as unvaccinated free-roaming dogs; dogs that are allowed to roam freely at any time, unsupervised, and therefore able to spread the virus through the population.

The lowest cost vaccine intervention for rabies is the static-point method, in which owners bring dogs to teams of vaccinators^5^. This is efficient in settings where dogs are well-cared for, but resulting coverages are often biased towards well-owned, fully confined dogs^6^. In communities that have sizeable proportions of free-roaming dogs (owned or unowned), this approach is unlikely to reach herd immunity in the at-risk dog population. Alternative vaccination methods such as door-to-door, capture-vaccinate-release, and oral vaccination have all been shown more effective at reaching at-risk dog populations, but are fraught with barriers such as costs, reliance on highly trained experts, and acceptability by communities and health officials^5,7^.

“Sink or source” is a common debate for many infectious diseases and has been a central debate among rabies scientists for decades^8^. The focus of rabies burden has traditionally centered on rural communities. In reality, dog-maintained rabies has been shown to impact a wide variety of communities, and outbreaks have been reported in urban, peri-urban, and rural settings^1,9^. Numerous studies have also shown that rabies transmission in wildlife reservoir species shows strong density dependence, which has led to interventions targeting the “source” of disease in high density populations^10,11^. Studies are conflicting on whether dog-maintained rabies is truly density dependent, which has also cast confusion over which community-types are the “source” for maintaining enzootic transmission, and which are the “sink” – communities where the virus spills into, experience cases, but transmission is not maintained^12,13^.

We describe an analysis using real-world surveillance data from Haiti’s National Rabies Surveillance Program to determine if there is a correlation between where rabies cases are detected and the free-roaming dog density^14^. The results can inform strategies for control, allocation of limited resources for interventions to where they will be most impactful, and improvements to transmission models.

## Methods

Haiti, a Caribbean nation of 11 million people largely of West African descent, operates an advanced rabies surveillance system that utilizes two mechanisms for alerts of suspected rabid animals: (1) Healthcare providers treating bite victims and (2) Reports of rabies-suspect animals directly from community members. The program was initiated in 2013 as a pilot and scaled up to near-national surveillance coverage as of 2018. From 2018 to 2023 the program routinely investigated over 2,000 suspected rabid animals (primarily dogs), annually. This program integrates veterinary and public health sectors under a One Health approach, employing trained surveillance officers to investigate animal bite incidents, conduct rabies testing, and facilitate timely post-exposure prophylaxis for bite victims^5,14^.

The country of Haiti was categorized into 20 urbanization levels characterizing the population density and road connectivity, at approximately 1 km^2^ scale, using the Settlement Type and Road Connectivity (STARC) methodology^15^. Hexagons of approximately 1 km^2^ were assigned a population density level of 1–6 using estimates from Meta’s High Resolution Population Density Maps from 2018 (1 for high density areas of over 5,000 people, to 6 for areas where no people live) and a road connectivity level of 1–4 (1 for connected via primary roads, to 4 for disconnected) using OpenStreetMap data^16^. These two factors were used in combination to describe the urbanization of each hexagon as S*X.Y*, where *X* is the population density level and *Y* is the road connectivity level. A full STARC map and complete breakdown of urbanization levels and community types can be found in Supplementary Fig. S1 and Table 1.

**Table 1:** Suspected animal rabies case investigations, Haiti 2018–2023 (66 months).

Urbanization level	Community type	High risk^+^		Moderate risk^+^		Low risk^+^		Negligible risk^+^		Total
		n**	%	*n*	%	*n*	%	*n*	%	
1.1	Urban	731 (19)	*17%*	167	*4%*	694	*16%*	2,669	*63%*	4,261
1.2	Urban	–	*0%*	2	*5%*	28	*64%*	14	*32%*	44
1.3	Urban	–	*0%*	–	*0%*	3	*33%*	6	*67%*	9
1.4	Urban	–		–		–		–		–
2.1	Urban	237 (29)	*7%*	292	*8%*	1,089	*30%*	1,969	*55%*	3,587
2.2	Urban	41 (1)	*5%*	32	*4%*	168	*22%*	507	*68%*	748
Urban total		**1**,**009 (49)**	***12%***	**493**	***6%***	**1**,**982**	***23%***	**5**,**165**	***59%***	**8**,**649**
2.3	Peri-urban	83 (3)	*11%*	85	*11%*	280	*38%*	293	*40%*	741
2.4	Peri-urban	–	*0%*	–	*0%*	7	*47%*	8	*53%*	15
3.1	Peri-urban	29 (10)	*8%*	22	*6%*	158	*41%*	176	*46%*	385
3.2	Peri-urban	12 (2)	*5%*	10	*4%*	88	*37%*	125	*53%*	235
3.3	Peri-urban	47 (7)	*4%*	77	*7%*	492	*44%*	501	*45%*	1,117
3.4	Peri-urban	13 (2)	*3%*	18	*4%*	147	*34%*	252	*59%*	430
Peri-urban total		**184 (24)**	***6%***	**212**	***7%***	**1**,**172**	***40%***	**1**,**355**	***47%***	**2**,**923**
4.1	Rural	-	*0%*	-	*0%*	7	*35%*	13	*65%*	20
4.2	Rural	1 (1)	*4%*	1	*4%*	11	*39%*	15	*54%*	28
4.3	Rural	-	*0%*	1	*2%*	24	*49%*	24	*49%*	49
4.4	Rural	3 (1)	*2%*	3	*2%*	56	*44%*	66	*52%*	128
5.1	Very rural	–	*0%*	–	*0%*	5	*100%*	–	*0%*	5
5.2	Very rural	–		–		–		–		–
5.3	Very rural	–	*0%*	–	*0%*	1	*25%*	3	*75%*	4
5.4	Very rural	–	*0%*	–	*0%*	1	*33%*	2	*67%*	3
Rural total		4 (2)***	***2%***	**5**	***2%***	**105**	***44%***	**123**	***52%***	**237**
Total		**1**,**197 (75)**	***10%***	**710**	***6%***	**3**,**259**	***28%***	**6**,**643**	***56%***	**11**,**809**

^***+***^high-risk, moderate risk, low risk and negligible risk animals were determined based on rabies probability classification model (Extreme Gradient Boosting) as published in Keshavamurthy et al. 2024. *910 investigations did not include location data and were removed from analysis (3.7% of all investigations). **(n) are the number of laboratory-confirmed animals within the high risk group. ***Confirmed sample from 4.2 was located 0.18 km from a peri-urban community. Confirmed sample 4.4 was located 0.32 km from a peri-urban community. A map showing the location of these confirmed cases in relation to STARC categories can be found in supplemental Figure  S2 . Statistically significant values (*p *< 0.05) are in bold.

Sixty-six months of routine animal rabies surveillance data (June 2018 – Dec 2023) collected by the Worldwide Veterinary Services, Rabies Exposure and Contact Tracing App (REACT) was geospatially overlayed with the STARC hexagonal map and aligned to urbanization categories to demonstrate surveillance effort and likely rabies case detection across this urban-rural spectrum^17^. Surveillance effort was defined as the number of case investigations per 10,000 people. Likely rabies cases were determined based on a previously published animal rabies prediction model^18^. In brief, we developed an Extreme Gradient Boosting (XGB) machine learning model to estimate the probability of rabies in animals investigated during routine surveillance. The model uses commonly collected case history and clinical signs data to generate a predicted probability, which is then used to classify animals into a four-tiered risk stratification framework: high, moderate, low, or negligible risk. This methodology provides highly accurate and reliable risk stratification. This prediction approach enhances the utility of case investigation data, providing more epidemiologically useful information than standard rabies clinical case definitions, particularly in settings lacking accessible laboratory services. All laboratory confirmed and high risk cases, in addition to 20% of moderate risk, 1% of low-risk animals, and 0% of negligible-risk animals were considered likely to have had true rabies virus infection and are referred to as “likely rabies cases”.

Rabies surveillance data is well-known to under-represent the true disease burden; for the purposes of this analyses we assumed a median case detection rate (CDR) of 5%, which assumes that for every 1 case detected through routine surveillance, 19 cases went undocumented^19^ Case investigations, adjusted for the number of people residing in the urbanization category, were calculated across the urbanization categories, and these were then adjusted to account for urbanization-level variations in surveillance effort through the equation: *[CIR/median(CIR*_*all*_)] * CDR, where CIR = Case Investigation Rate per Human Capita. The resulting CDR adjustment factor was then applied to the number of likely rabies cases to account for under-detection in communities with fewer investigations through the equation, *(Likely Rabies Cases)/(CDR Adjustment Factor)*. A sensitivity analysis was conducted by varying the CDR to a low of just 1% and a high of 10%, which correspond to weak and adequate surveillance systems, respectively^20^.

Annualized across 66 months of routine surveillance data, likely rabies cases were divided by the CDR adjustment factor to derive the estimated total number of dog-mediated rabies cases in each urbanization level, assuming an enzootic state of transmission. The effective reproduction number (Re) of rabies virus infection was estimated using the previously published Rabies Economics model, where each of the 20 urbanization levels were modeled and the Re was adjusted until the prediction matched the expected number of annual rabid animals, defined as the detected rabid dogs adjusted for a 1%, 5%, and 10% CDR^21,22^ (Table 2, Table S1). Prediction values were defined as the annual average number of model-derived dog rabies cases from program year 20 through 30 of the model estimates, which reflects an enzootic transmission state. Routine dog vaccination was not maintained after COVID-19 lockdowns were implemented nor after the societal instability that began in 2020, therefore vaccination coverage of the free-roaming dog population was estimated to be as follows Urban: 50%, Peri-Urban: 30%, Rural 10%, Very Rural 0%^23,24^.

**Table 2:** Haiti animal rabies surveillance effort, investigation results, and calculated reproductive rate of infection.

Urbanization level	Area (km2)	Human population (2019)	Free-roaming dog (FRD) population*	Suspect rabies cases investigated annually**	Case investigation rate (per 10,000 people)	Case investigation rate - adjustment factor***	Likely annual rabid animals detected****	Likely annual rabid FRD, adjusted	Re *****	Median rabies rate (per 1,000 FRD)
1.1	841	4,615,573	181,908	775	1.7	10.8%	140.2	1,300	1.67	14.3
1.2	70	164,336	8,217	8	0.5	3.1%	0.12	4	1.59	0.97
1.3	45	76,538	8,610	2	0.2	1.7%	0.005	0.3	1.57	0.07
1.4	6	7,842	1,008	0	0	0.0%	*na*	*na*	*na*	*na*
2.1	1,433	1,668,525	121,802	652	3.9	25.1%	55.7	222	1.64	1.82
2.2	689	645,788	46,966	136	2.1	13.5%	8.9	66	1.63	1.40
Urban	**3**,**084**	**7**,**178**,**602**	**368**,**511**	**1**,**573**	**2.2**	**na**	**204.9**	**1**,**592**	**1.62**	**1.4**
2.3	1,161	945,131	77,329	135	1.4	9.2%	18.7	204	1.27	2.64
2.4	345	268,066	21,933	3	0.1	0.7%	0.01	2	1.22	0.08
3.1	1,299	362,696	45,337	70	1.9	12.4%	6.4	52	1.26	1.14
3.2	988	250,264	25,861	43	1.7	11.0%	2.7	24	1.25	0.95
3.3	2,676	628,585	94,288	203	3.2	20.7%	12.2	59	1.26	0.62
3.4	1,726	343,705	61,867	78	2.3	14.6%	3.3	23	1.25	0.37
Peri-urban	**8**,**195**	**2**,**798**,**447**	**326**,**615**	**532**	**1.9**	**na**	**43.3**	**363**	**1.25**	**0.79**
4.1	897	128,839	14,725	4	0.3	2.0%	0.01	0.7	1.02	0.04
4.2	634	89,239	7,764	5	0.6	3.6%	0.06	2	1.02	0.20
4.3	1,725	228,190	25,671	9	0.4	2.5%	0.08	3	1.03	0.12
4.4	1,386	167,738	18,870	23	1.4	8.8%	0.94	11	1.04	0.56
5.1	566	10,031	4,012	1	0.9	6.4%	0.009	0.1	< 0.99	0.04
5.2	526	9,782	4,255	0	0	0.0%	*na*	*na*	*na*	*na*
5.3	1,980	34,351	15,458	1	0.2	1.9%	0.002	0.1	< 0.99	0.01
5.4	8,136	44,983	20,242	1	0.1	1.4%	0.002	0.1	< 0.99	0.01
Rural	**15**,**850**	**713**,**153**	**110**,**997**	**44**	**0.6**	**na**	**1.1**	**16.4**	**0.80**	**0.04**
Total	**27**,**129**	**10**,**690**,**202**	**806**,**123**	**2**,**149**	**2.0**	**na**	**249**	**1**,**972**	**na**	**0.42**

*Dog population estimates were obtained from 61 dog enumerations studies conducted across Haiti. Further details can be found in figure S3. **Annualized to reflect average investigation rate across the 66-month study period. ***Adjusted to fit median case detection rate for entire population of 1%. ****20% of Moderate-risk and 1% of Low-risk rabies cases were included to account for the likelihood of true rabies cases being classified into these categories. *****Free roaming dog vaccination coverages were based on historical vaccination practices: Urban (50%), Peri-Urban (30%), Rural level 4 (10%), and Rural level 5 (0%). Average dog lifespan set to 3 years and dog birth rate (per 1,000) set to 750. *Road connectivity was defined as: “Well Connected” = x.1, “Moderately Connected” = x.2, “Poorly Connected” = x.3 and x.4. Urbanicity was defined as previously described in the methods. Statistically significant values (*p* < 0.05) are in bold.

Rabies incidence rates, Re and suspected human rabies exposures were calculated across the urbanization levels and compared to the free-roaming dog density. STARC-stratified free-roaming dog densities and human-to-dog ratios were estimated using the methodology described by Moran et al. using data obtained from 61 post-vaccination campaign and dog population surveys conducted from 2015 to 2019^25^. Urban, peri-urban, and rural dog-mediated rabies incidence rates were calculated within the respective urbanization categories (Table 2). Mid-P exact tests were conducted to obtain the median incidence rate and 95% confidence interval.

The relationships between free-roaming dog (FRD) density and three rabies-related outcomes (Re, reported dog bite rate per 10,000 human population, and the CDR-adjusted rabies rate per 1,000 FRD) were evaluated using two statistical models: log-anchored linear and piecewise linear. The analysis was repeated using susceptible FRD (unvaccinated) in place of total FRD. Log-anchored models were fitted using log-transformed FRD density, constrained to pass through the origin (0,0) to reflect the biological assumption that zero dogs results in zero risk. Piecewise models allowed separate slopes above and below an optimized breakpoint. Gaussian (identity-link) models were applied to Re, and Gamma (log-link) generalized linear models were used for rabies incidence and bite-rate outcomes to account for there distributional properties. It was not possible to obtain an exact Re value for STARC-stratification levels with a Re of < 1, therefore the median of the possible Re range of 0–0.99 was assigned. The best model based on model fit was identified based on Akaike Information Criterion (AIC). Model performance was assessed using centered R², root mean squared error (RMSE) and mean absolute error (MAE). To enable comparison across differently scaled outcomes, we also computed relative RMSE (RRMSE) and categorized fit quality as excellent, good, or poor based on scaled error thresholds. The 95% confidence interval at which Re falls below 1.0 was calculated for all relevant models. Median rabies incidence rates and case investigations rates were calculated for the combination of urbanization category and road connectivity.

## Results

A total of 11,809 suspect animal rabies cases were investigated by Haiti’s rabies program over 66 months spanning 2018–2023. Suspect animal rabies cases, which typically represent at least one exposed human under Haiti’s surveillance approach, were primarily reported from urban communities (average of 1,573 per year, 73% of all case investigations) (Table 1). An additional 531 annual investigations occurred in peri-urban communities (25%) and only 2% in rural-defined settings. The likelihood of these exposures being due to a rabid animal followed a similar trend, with 12% of urban exposures, 6% of peri-urban exposures, and less than 1% of rural exposures occurring from a likely rabid animal. Overall, an estimated 1,972 rabid dogs are estimated to die in Haiti each year; 0.2% of the free-roaming dog population (Table 2). Assuming a 33% annual population turnover rate in free-roaming dogs (*n* = 266,021)^20^, the results of this study suggest that 0.7% of free-roaming dog deaths are due to rabies virus infection (Table 2).

**Fig. 1:**
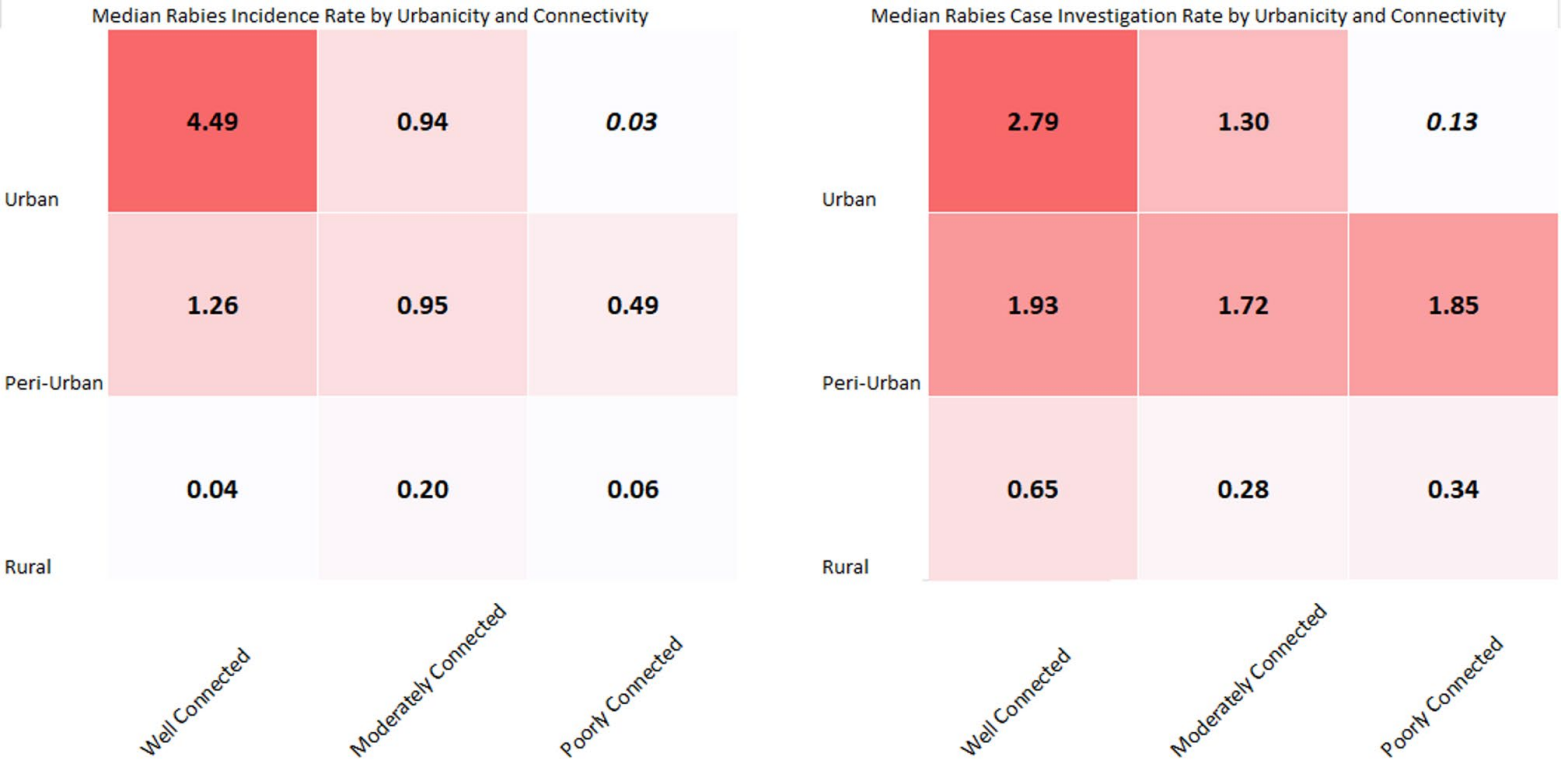
Median rabies investigation and incidence rates by urbanization and road connectivity.

**Fig. 2:**
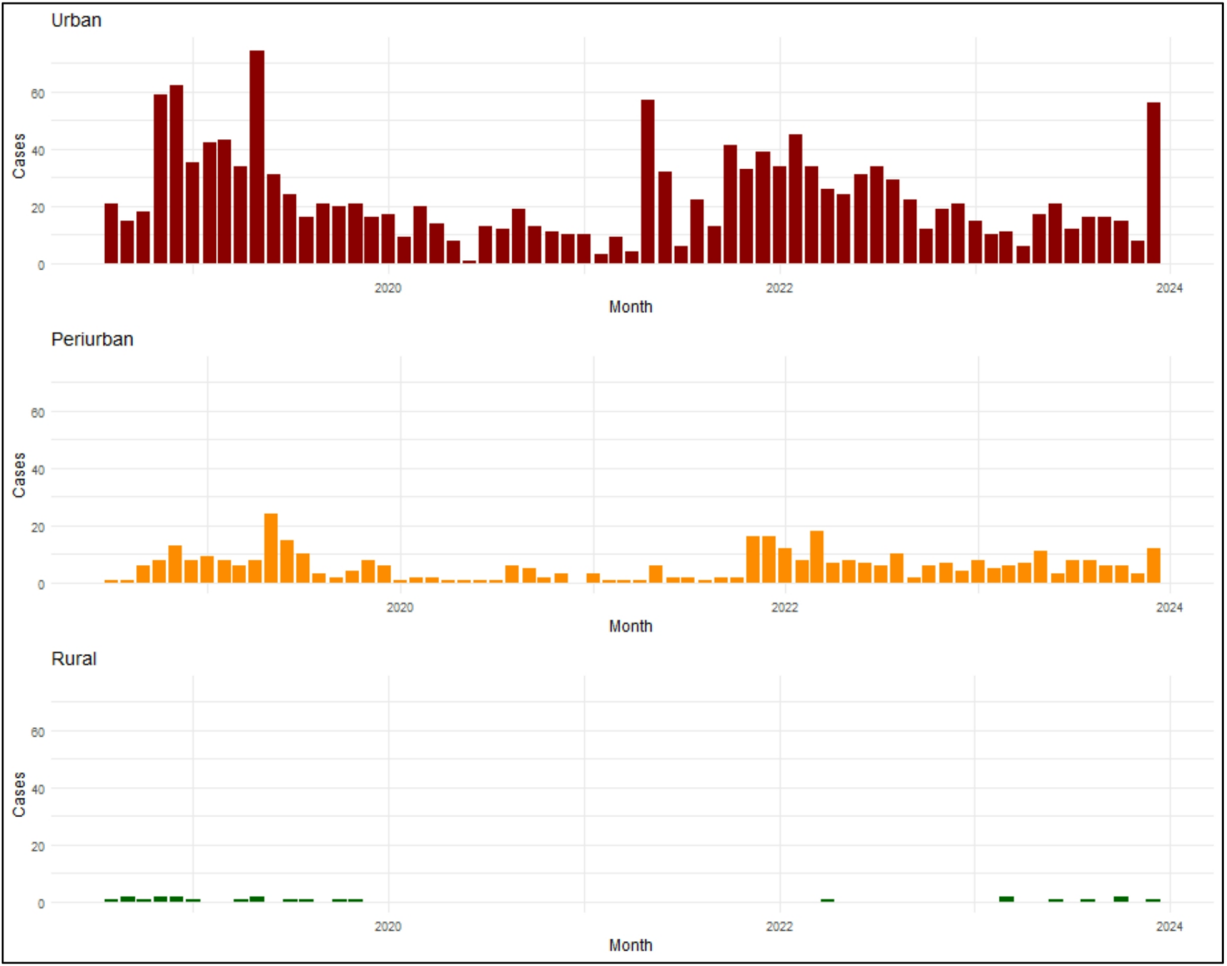
Likely rabies cases detected in urban, peri-urban, and rural communities by month, 2018–2023.

Rabies case investigations were conducted in all but two urbanization levels (1.4 and 5.2, Table 1). Most investigations (64%) were conducted in 1.1 and 2.1 (urban) communities, where human and dog population densities were comparably high (Table 1). Rabies CIRs varied by urbanization level, with the highest rate of investigation reported from levels 2.1 and 3.3 (urban and peri-urban) and lowest case investigation rates occurring in 2.4 and 5.4 (peri-urban and rural) (Fig. 1). Animals likely infected with rabies virus were detected in all urbanization levels where investigations were conducted, however the average annual number of these cases varied (range < 1–149 per year). Likely rabid animals were consistently detected in urban and peri-urban communities, while rural communities had sporadic detections (Fig. 2).

After adjusting for variation in surveillance effort (CIRs) and the standard 5% CDR, likely rabid animals were most found in urbanization level 1.1 (highly urbanized, 1,300 per year) (Table 2). Likely rabid animals were less common in communities that were disconnected (X.4) and rural (4.Y and 5.Y) (Table 2; Fig. 1). When considering dog-mediated rabies incidence rates per 1,000 free-roaming dogs, urban communities had a median incidence rate of 1.4, followed by peri-urban communities (0.8) and then rural (0.04) (Table 2; Fig. 2).

The highest adjusted rabies incidence rates were in the most urbanized communities (1.1, 2.3, and 2.1), and high incidence rates were also reported in several peri-urban communities (2.2, 3.1, and 3.2) (Table 2). Rural communities had low adjusted rabies incidence rates. Associations between rabies incidence rates and free-roaming dogs showed poor fit by both piecewise models (R^2^ = 0.67, RRMSE = 1.3) and log-anchored models (R^2^ = 0.02, RRMSE = 1.73). Similarly, poor associations were noted between free-roaming dog density and the incidence rate of reported dog bites (piecewise models: R^2^ = 0.52, RRMSE = 0.8, log-anchored model: R^2^ = 0.05, RRMSE = 0.96) (Table S2).

**Fig. 3:**
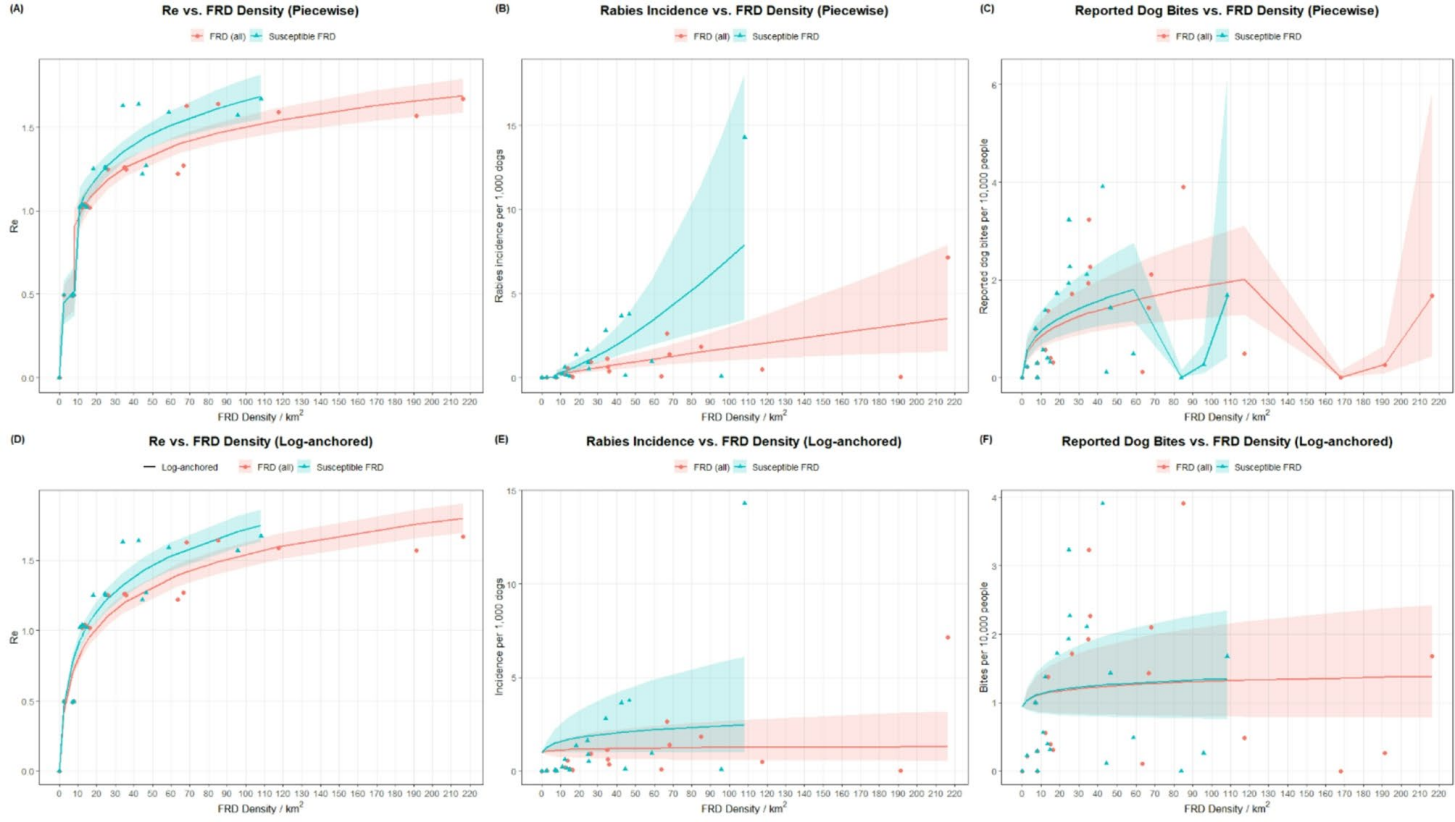
Relationships between free-roaming dog (FRD) density and rabies-related outcomes (i) effective reproduction number (Re), (ii) rabies incidence rate among dogs, and (iii) reported dog-bite rate in humans are shown for two model classes adjusted for a case detection rate of 5%. Piecewise models (top row) capture potential threshold behavior via breakpoints on FRD, whereas log-anchored models (bottom row) assume a continuous logarithmic relationship passing through FRD = 0. Shaded ribbons represent 95% confidence intervals around fitted means for FRD (all dogs) (red) and rabies-susceptible FRD (blue).

The Re was highest in urban communities, ranging from 1.57 to 1.67 (Table 2). All peri-urban communities had Re values greater than 1.0, ranging from 1.22 to 1.27. All rural communities had Re values near or below 1.0, suggesting that sustained maintenance of rabies virus may not be possible in these low free-roaming dog-density settings (range 1.04 – <0.99) (Table 2; Fig. 3). The Re value showed a strong association with the free-roaming dog density using both disconnected piecewise models (R^2^ = 0.96, RRMSE = 0.08) and log-anchored (R^2^ = 0.9, RRMSE = 1.2). Based on disconnected piecewise model of FRD density and Re, rabies would likely not become enzootic if the FRD density was below 12.2 (95% Confidence Interval: 11.3–16.3). A detailed list of all the model-estimated FRD density (95% CI) where Re is equal to 1 is presented in Table S3.

## Discussion

Numerous studies have shown that rabies virus, along with many infectious diseases, follows density-dependent transmission^26,27^. However, many of these studies are focused on wildlife, which are unrestricted by fences, leashes, and homes. Similar studies on dog populations have shown weaker density dependence, yet many of these studies fail to account for the roaming nature of dogs^28^. Rabies virus is primarily transmitted through direct contact, typically a bite, therefore in the context of domesticated dogs, those that have movement restrictions such as impermeable fences and leash-walking are at very low risk of becoming exposed and contributing to enzootic transmission cycles. When considering only free-roaming dogs in a transmission dynamics analysis, critical assumptions about population mixing are better satisfied. In our analysis, where only free-roaming dogs were considered, we found strong evidence for density-dependent transmission, with most of the viral maintenance and high-risk human rabies exposures occurring in communities with relatively high densities of free-roaming dogs.

“Sink versus source” is an important epidemiologic feature of infectious diseases, particularly when trying to determine the nidus of infection, identify high-risk populations, and allocating effective control resources. Dog-maintained rabies virus transmission has often been characterized as a “rural disease”, and there are numerous records of outbreaks occurring in communities that would colloquially be considered “rural”^1,9^. However, there is no clear evidence as to whether rural rabies outbreaks represent enzootic transmission versus periodic introductions from a nearby source, and there is no generally agreed upon definition of “rural” to base these epidemiologic observations.

The analysis conducted here, reflecting data from an advanced surveillance system in a dog-maintained rabies endemic setting, strongly suggests that urban and peri-urban communities with high densities of free-roaming dogs are the likely source of enzootic rabies virus in Haiti. However, despite the strong evidence for density-dependence, rare rabies cases were still detected in very rural settings. These cases appeared to be sporadic, possibly through the translocation of infected dogs resulting in limited transmission in these rural landscapes. It is important to note that the urbanization levels used in this analysis are not necessarily contiguous areas. Dogs may roam from where case investigations were conducted into areas classified as different urbanization levels, but we expect there is minimal travel across levels based on the typical home range of a dog. Results of this study show rare and intermittent cases in rural communities juxtaposed to relatively common and consistent detection of rabies cases in higher density communities. Improving access to genetic characterization of rabies viruses could inform the origin of rabies outbreaks, particularly when they occur in low dog-density communities.

Connectedness of communities also may play a role in the introduction and maintenance of rabies virus in dog populations. Despite high dog densities, incidence rates of dog-mediated rabies and Re were drastically lower in disconnected, high-density communities of 1.4, 2.4, and 3.4 (Fig. 1). This may suggest that maintaining dog-mediated rabies requires not only a minimum density threshold, but also a population that is well-connected to other enzootic communities. Being disconnected from other communities may also protect from natural and human-mediated translocation of rabid animals. Enhancing investigations to try to identify index cases and their origin should be pursued to better understand the role of rural rabies outbreaks and viral maintenance.

The strong correlation between free-roaming dog density and Re may not always correlate to urban and rural dichotomies. Dog ownership rates and practices of confinement vary drastically between communities, cultures, and countries^29,30^. In Haiti, despite having 1 dog for every 2 people in rural settings, the free-roaming dog density is quite low. Other countries may not have similar dog population characteristics, which could lead to different observations as to the association between urbanicity and rabies transmission. For example, many upper and middle-income countries have very few free-roaming dogs in urban centers, often reflecting advances in dog population management programs. In these settings of high urbanicity but low free-roaming dog density, we would not expect rabies virus transmission to be maintained.

Interestingly, when examining the relationships between Re and free-roaming dog density, the rabies virus reproduction number falls below 1.0 in settings where there are fewer than 10 free-roaming dogs per square kilometer. This finding should be re-examined in other settings, particularly where dog ownership practices differ from those of Haiti. In countries with similar cultures of dog ownership, this critical finding may help inform which communities are sources of rabies infection, and therefore should be prioritized for interventions such as vaccination, education, and dog population management. While it is best practice to vaccinate every dog in every community against rabies, this is not practical in low- and middle-income countries, and scientifically based methods for maximizing impact with limited vaccine resources should be considered.

Another possible application of the density-dependence findings presented here could be a re-envisioning of dog vaccination program goals. Similar to an impermeable fence, vaccination acts as a biological barrier to the virus. Therefore, rather than necessarily striving for 70% vaccination coverage, which is recommended by the World Health Organization, it may be advantageous and resource-saving for countries to consider approaches to vaccinate enough dogs to get below 10 unvaccinated (e.g. rabies susceptible) free-roaming dogs per square kilometer. Planning vaccination campaigns through this approach would likely lead to community-tailored vaccination coverage goals. Numerous studies, including those that were considered in the model that established the 70% herd immunity statistic, have shown that a wide variety of coverages were successful at eliminating dog-maintained rabies^4,31^. Some reports demonstrated that as low as 40% herd immunity can be effective in certain populations^32^. Perhaps a better understanding of the dynamics between a critical density threshold (e.g., 10 dogs per km^2^) and vaccination coverage can lead to community-tailored vaccination goals.

Caution should be exercised when attempting to interpret these findings in relation to dog population management. Many high-income countries have eliminated rabies through a combination of vaccination programs and the humane removal of unwanted free-roaming dogs, typically through shelter medicine programs. However, low- and middle-income countries often struggle to sustain adequate resources necessary to implement humane dog population management actions. When free-roaming dog populations become overwhelming to a community or present a public health danger, ineffective culling operations are often proposed as a short-term solution. Indiscriminate dog culling has been proven-ineffective at eliminating rabies and can damage important relationships with community members that are relied upon to participate in rabies vaccination and surveillance programs.

Given the vast heterogeneity in cultures of dog ownership and unique settings in which dogs and humans cohabitate, there likely is no singular transmission model that can reflect such broad diversity. The results here reflect not just what can be gleaned when a country invests in advanced rabies surveillance and dog population studies, but also provides strong evidence that free-roaming dog density is a strong driver of enzootic dog-maintained rabies.

## Supplementary Information

Below is the link to the electronic supplementary material.

Supplementary Material 1

Supplementary Material 2

## Data Availability

All data files used to conduct this analysis can be shared upon request by the corresponding author, but after all patient identifying information (PII), including GPS points if they are deemed to reflect a bite victim’s residence is removed.
