# Supplementary material for: Higher free-roaming dog density sustains rabies virus transmission in Haiti
Source: Sci Rep. 2026 Jan 24;16:5543. doi: 10.1038/s41598-026-35359-y (PMC12886927; doi:10.1038/s41598-026-35359-y)
Supplement: Supplementary file 2 — Supplementary Material 2 [file 41598_2026_35359_MOESM2_ESM.docx]

**Supplemental Material**

**Figure S1:** STARC distributions across Haiti, 2019

**
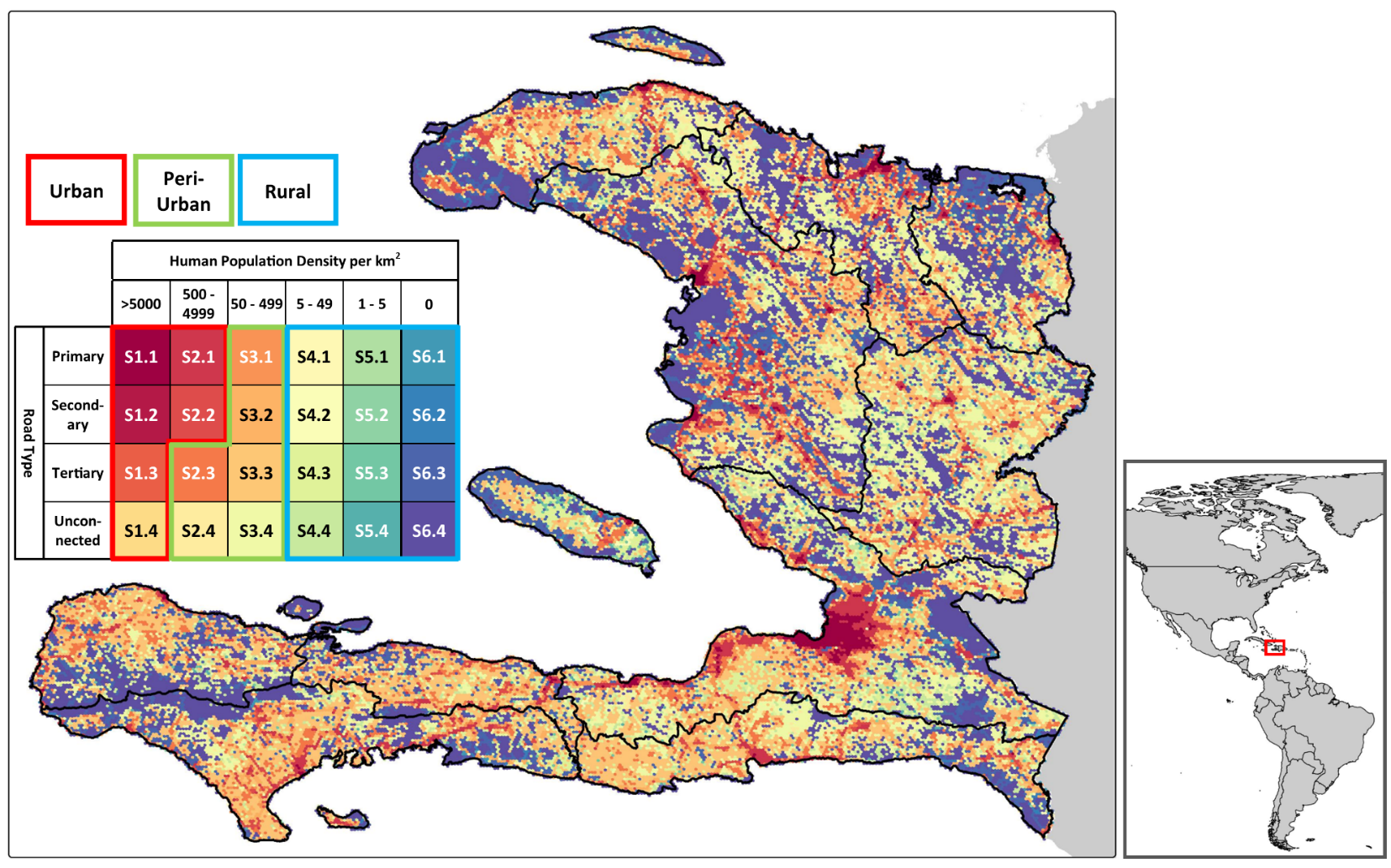
**

*Map created using QGIS version 3.22.0. National and sub-national boundaries obtained from Natural Earth and geoBoundaries.

**Figure S2: Map showing the location of these confirmed cases in relation to STARC categories**

**
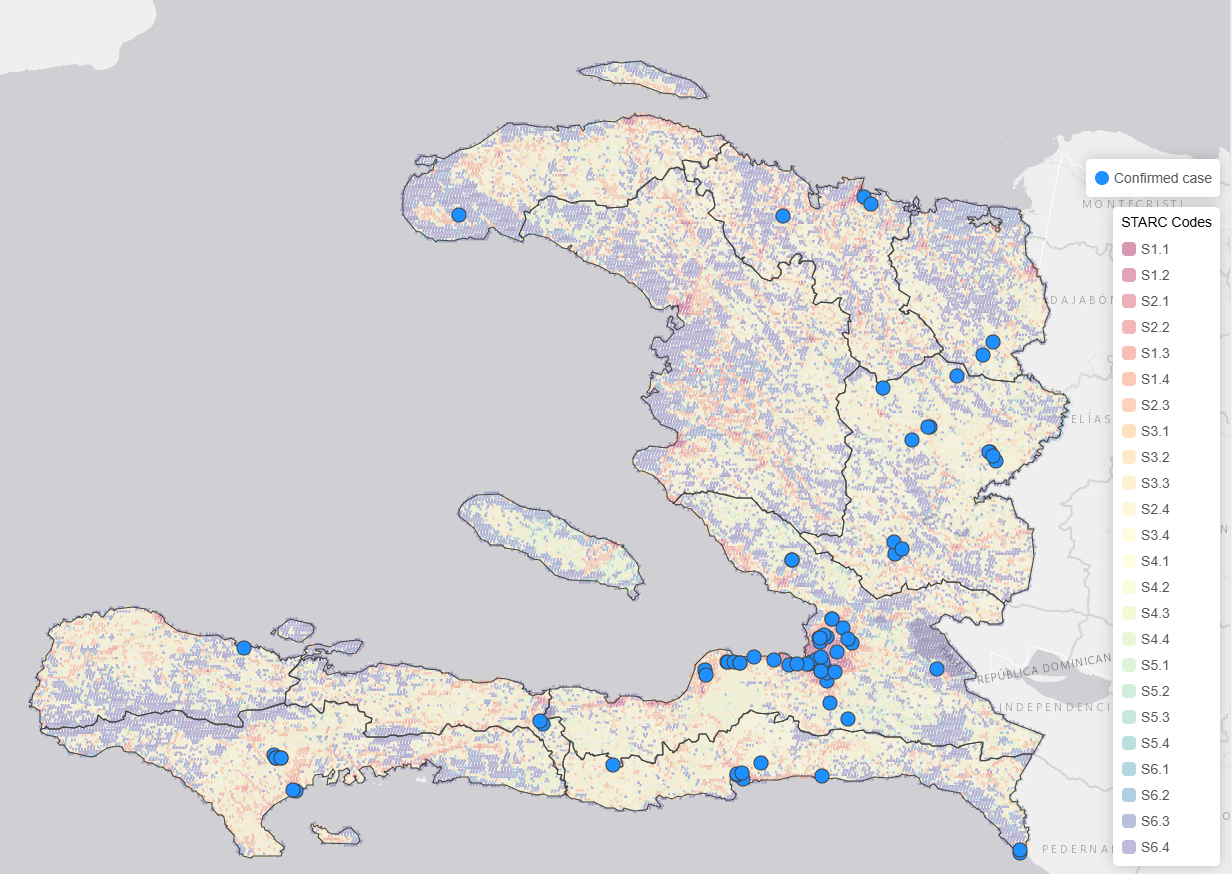
**

**Figure S3:** From 2015 – 2019, 61 dog population surveys were conducted across a broad spectrum of communities in Haiti. Surveys included door-to-door and field-based observation studies. Lincoln-Petersen with the Wallace free-roaming dog adjustment were applied ^21^. Communities were characterized based on population density and road connectivity, as described in the methods section. Human-to-Dog ratios were obtained from this survey data and median and quartile values were obtained. This data, found in Table S2, was then used to estimate the free roaming dog population used in this analysis.

**
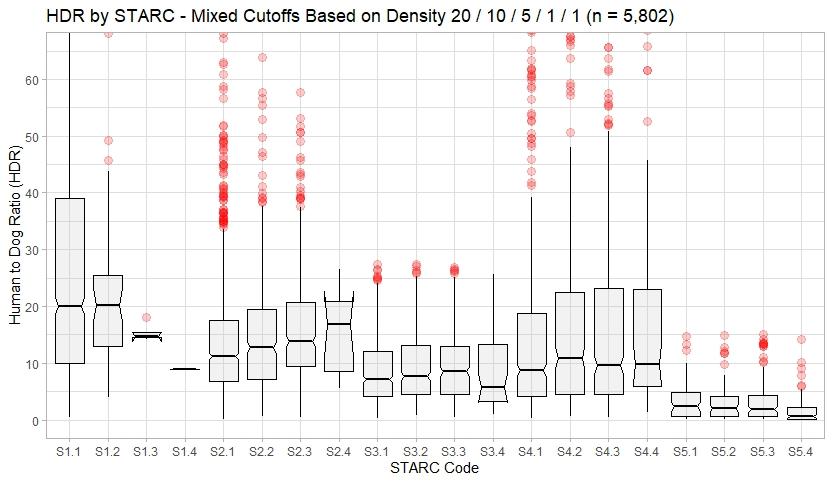
**

**Table S1: Model Parameters and Distributions for Designing Effective Vaccination Methods, Haiti 2022 (Rabies Econ)**

|  | **Items** | **Parameters/Distributions** | **Source** |
| --- | --- | --- | --- |
| **Re Model** | Bite Rate - S1 | 1% | [Fenelon](https://www.cambridge.org/core/journals/epidemiology-and-infection/article/knowledge-attitudes-and-practices-regarding-rabies-risk-in-community-members-and-healthcare-professionals-petionville-haiti-2013/E0D4C888976847539526D08C0036CBC1) |
|  | Bite Rate - S2 | 2% | [Schildecker](https://onlinelibrary.wiley.com/doi/10.1111/tbed.12531) |
|  | Bite Rate - S3, S4, S5 | 4% |  |
|  | Average Free Roaming Dog Density | Variable based on STARC Code | Data-derived |
|  | Analysis Derived Re | 0.19 * LN(FRD Density) + 0.54 | Data-derived |
| **Rabies Econ Model** | Initial Re | Variable (see methods) | User Defined |
|  | Spay/Neuter Proportion | 0% | User Defined |
|  | Probability of Receiving PEP - S1 | 90% | [Etheart et. al.](https://www.thelancet.com/journals/langlo/article/PIIS2214-109X(17)30321-2/fulltext) |
|  | Probability of Receiving PEP - S2 | 90% |  |
|  | Probability of Receiving PEP - S3 | 75% |  |
|  | Probability of Receiving PEP - S4 | 66% |  |
|  | Probability of Receiving PEP - S5 | 50% |  |
|  | Lead-in Vaccination Coverage - Year 1 | 5% | [Wallace et. al.](https://www.frontiersin.org/articles/10.3389/fvets.2017.00009/full) |
|  | Lead-in Vaccination Coverage - Year 2 | 5% |  |
|  | Lead-in Vaccination Coverage - Year 3 | 20% |  |
|  | Lead-in Vaccination Coverage - Year 4 | 20% |  |
|  | Lead-in Vaccination Coverage - Years 5 - 20 | 30% (Peri Urban) / 50% (Urban) |  |
|  | All other values | Unchanged - See supplemental | [Kunkel et. al.](https://www.nature.com/articles/s41598-021-92067-5) |
| **Vax-PLAN Model** | Parenteral Vaccine Efficacy | 100% | Default / User Defined |
|  | GDREP Phase | IIb | [Monroe et. al.](https://www.frontiersin.org/articles/10.3389/fpubh.2021.757668/full) |
|  | Vaccinator Capacity - SX.1 & SX.2 (b) | Central Point: 30 | [Undurraga et. al](https://www.sciencedirect.com/science/article/pii/S0264410X20307672?via%3Dihub) |
|  |  | Door to Door: 50 |  |
|  |  | Capture-Vaccinate-Release: 25 |  |
|  | Vaccinator Capacity - SX.3 & SX.4 (b) | Central Point: 20 |  |
|  |  | Door to Door: 40 |  |
|  |  | Capture-Vaccinate-Release: 15 |  |
|  | Vaccination Campaign Duration | 10 days | Default / User Defined |
|  | All other values | Unchanged - See supplemental | [Wallace et. al.](https://www.cambridge.org/core/journals/epidemiology-and-infection/article/estimating-the-effectiveness-of-vaccine-programs-in-dog-populations/E0D25E5DFB352731121EDBA5DEFEF7EE) |
| *(a) Re - Effective Reproduction Number*  *(b) PEP - Post-Exposure Prophylaxis (c) Vaccinator Capacity expressed as the number of dogs vaccinated, per vaccinator, per vaccination day (d) Vaccination coverage in Rural areas was fixed at 10% for all years* | | | |

**Table S2: Model forms and fit statistics for the association between free-roaming dog (FRD) density and epidemiologic outcomes (Re, rabies incidence, and reported dog bites), stratified by case-detection rate (CDR) scenario.** Analysis used case detection rate (CDR) scenarios (1 %, 5 %, 10 %) adjusted for the full free-roaming dog population and subset of dogs susceptible to rabies infection for sensitivity analysis

| **Type** | **Outcome** | **Model** | **MAE** | **RSME** | **R2** | **RRMSE** | **AIC** | **Interpretation** | **Equation** |
| --- | --- | --- | --- | --- | --- | --- | --- | --- | --- |
| CDR 1% (All) | Re | Log-Anchored Model | 0.12 | 0.14 | 0.87 | 0.12 | -15.17 | Excellent fit | 0.3415 * ln(1 + FRD) |
|  |  | Piecewise model (discontinuous GLM) | 0.06 | 0.09 | 0.94 | 0.08 | -23.84 | Excellent fit | ln(FRD) ≤ 2.055: 0.4966 + -0.002*ln(FRD)  //  ln(FRD) > 2.055: 0.8923 + 0.2567*(ln(FRD) − 2.055) |
|  | Incidence | Log-Anchored Model | 4.24 | 7.24 | 0.74 | 1.48 | 82.88 | Poor fit | E[y] = (1 + FRD)^0.40748 − 0.0173 |
|  |  | Piecewise model (discontinuous GLM) | 2.15 | 3.98 | 0.89 | 0.81 | 70.56 | Poor fit | ln(FRD) ≤ 4.765: -3.645 + 1.3478*ln(FRD)  //  ln(FRD) > 4.765: -22.5147 + 42.6888*(ln(FRD) − 4.765) |
|  | Bites | Log-Anchored Model | 0.88 | 1.07 | 0.09 | 0.87 | 50.67 | Poor fit | E[y] = (1 + FRD)^0.07576 − 0.056 |
|  |  | Piecewise model (discontinuous GLM) | 0.67 | 0.95 | 0.54 | 0.78 | 42.12 | Poor fit | ln(FRD) ≤ 4.765: -1.4423 + 0.5202*ln(FRD)  //  ln(FRD) > 4.765: -7.7647 + 13.5876*(ln(FRD) − 4.765) |
| CDR 10% (All) | Re | Log-Anchored Model | 0.13 | 0.14 | 0.86 | 0.12 | -15.60 | Excellent fit | 0.3322 * ln(1 + FRD) |
|  |  | Piecewise model (discontinuous GLM) | 0.06 | 0.09 | 0.94 | 0.08 | -25.58 | Excellent fit | ln(FRD) ≤ 2.055: 0.4966 + -0.002*ln(FRD)  //  ln(FRD) > 2.055: 0.8917 + 0.2368*(ln(FRD) − 2.055) |
|  | Incidence | Log-Anchored Model | 0.64 | 0.87 | 0.05 | 1.98 | -1.43 | Poor fit | E[y] = (1 + FRD)^-0.11964 − 0.00173 |
|  |  | Piecewise model (discontinuous GLM) | 0.26 | 0.46 | 0.73 | 1.04 | -27.23 | Poor fit | ln(FRD) ≤ 4.765: -6.292 + 1.4381*ln(FRD)  //  ln(FRD) > 4.765: -17.4623 + 30.1474*(ln(FRD) − 4.765) |
|  | Bites | Log-Anchored Model | 0.89 | 1.07 | 0.06 | 0.92 | 51.57 | Poor fit | E[y] = (1 + FRD)^0.06802 − 0.056 |
|  |  | Piecewise model (discontinuous GLM) | 0.67 | 0.96 | 0.49 | 0.82 | 43.95 | Poor fit | ln(FRD) ≤ 4.765: -1.7175 + 0.5895*ln(FRD)  //  ln(FRD) > 4.765: -7.7647 + 13.5876*(ln(FRD) − 4.765) |
| CDR 5% (All) | Re | Log-Anchored Model | 0.12 | 0.14 | 0.90 | 0.12 | -17.20 | Excellent fit | 0.3346 * ln(1 + FRD) |
|  |  | Piecewise model (discontinuous GLM) | 0.06 | 0.09 | 0.96 | 0.08 | -28.22 | Excellent fit | ln(FRD) ≤ 2.055: 0.3982 + 0.0568*ln(FRD)  //  ln(FRD) > 2.055: 0.9017 + 0.2368*(ln(FRD) − 2.055) |
|  | Incidence | Log-Anchored Model | 1.09 | 1.61 | 0.02 | 1.73 | 27.65 | Poor fit | E[y] = (1 + FRD)^0.05149 − 0.00346 |
|  |  | Piecewise model (discontinuous GLM) | 0.70 | 1.24 | 0.67 | 1.34 | 8.12 | Poor fit | ln(FRD) ≤ 2.055: -4.2777 + 0.2066*ln(FRD)  //  ln(FRD) > 2.055: -1.7303 + 0.9005*(ln(FRD) − 2.055) |
|  | Bites | Log-Anchored Model | 0.90 | 1.06 | 0.05 | 0.96 | 51.76 | Poor fit | E[y] = (1 + FRD)^0.06802 − 0.056 |
|  |  | Piecewise model (discontinuous GLM) | 0.65 | 0.89 | 0.52 | 0.80 | 41.41 | Poor fit | ln(FRD) ≤ 4.765: -0.8697 + 0.3347*ln(FRD)  //  ln(FRD) > 4.765: -7.7647 + 13.5876*(ln(FRD) − 4.765) |
| CDR 1% (Susceptible) | Re | Log-Anchored Model | 0.13 | 0.17 | 0.82 | 0.14 | -9.68 | Excellent fit | 0.3801 * ln(1 + FRD) |
|  |  | Piecewise model (discontinuous GLM) | 0.08 | 0.12 | 0.91 | 0.10 | -15.88 | Excellent fit | ln(FRD) ≤ 2.055: 0.4966 + -0.002*ln(FRD)  //  ln(FRD) > 2.055: 0.919 + 0.3137*(ln(FRD) − 2.055) |
|  | Incidence | Log-Anchored Model | 7.82 | 14.32 | 0.84 | 1.66 | 97.74 | Poor fit | E[y] = (1 + FRD)^0.59457 − 0.0173 |
|  |  | Piecewise model (discontinuous GLM) | 3.47 | 6.40 | 0.94 | 0.74 | 83.58 | Poor fit | ln(FRD) ≤ 4.072: -4.7004 + 1.9517*ln(FRD)  //  ln(FRD) > 4.072: -22.0524 + 43.0661*(ln(FRD) − 4.072) |
|  | Bites | Log-Anchored Model | 0.90 | 1.07 | 0.05 | 0.92 | 51.74 | Poor fit | E[y] = (1 + FRD)^0.07215 − 0.056 |
|  |  | Piecewise model (discontinuous GLM) | 0.71 | 0.99 | 0.46 | 0.85 | 44.86 | Poor fit | ln(FRD) ≤ 4.072: -1.9117 + 0.7185*ln(FRD)  //  ln(FRD) > 4.072: -7.7647 + 13.5876*(ln(FRD) − 4.072) |
| CDR 10% (Susceptible) | Re | Log-Anchored Model | 0.13 | 0.14 | 0.86 | 0.12 | -15.60 | Excellent fit | 0.3322 * ln(1 + FRD) |
|  |  | Piecewise model (discontinuous GLM) | 0.06 | 0.09 | 0.94 | 0.08 | -25.58 | Excellent fit | ln(FRD) ≤ 2.055: 0.4966 + -0.002*ln(FRD)  //  ln(FRD) > 2.055: 0.8917 + 0.2368*(ln(FRD) − 2.055) |
|  | Incidence | Log-Anchored Model | 1.10 | 1.63 | 0.00 | 1.89 | 22.93 | Poor fit | E[y] = (1 + FRD)^0.02931 − 0.00173 |
|  |  | Piecewise model (discontinuous GLM) | 0.32 | 0.68 | 0.73 | 0.79 | -0.43 | Poor fit | ln(FRD) ≤ 4.765: -6.3479 + 1.5626*ln(FRD)  //  ln(FRD) > 4.765: -24.355 + 43.0661*(ln(FRD) − 4.765) |
|  | Bites | Log-Anchored Model | 0.89 | 1.07 | 0.06 | 0.92 | 51.57 | Poor fit | E[y] = (1 + FRD)^0.06802 − 0.056 |
|  |  | Piecewise model (discontinuous GLM) | 0.67 | 0.96 | 0.49 | 0.82 | 43.95 | Poor fit | ln(FRD) ≤ 4.765: -1.7175 + 0.5895*ln(FRD)  //  ln(FRD) > 4.765: -7.7647 + 13.5876*(ln(FRD) − 4.765) |
| CDR 5% (Susceptible) | Re | Log-Anchored Model | 0.12 | 0.16 | 0.88 | 0.14 | -12.22 | Excellent fit | 0.3725 * ln(1 + FRD) |
|  |  | Piecewise model (discontinuous GLM) | 0.07 | 0.11 | 0.94 | 0.10 | -19.58 | Excellent fit | ln(FRD) ≤ 2.055: 0.3982 + 0.0568*ln(FRD)  //  ln(FRD) > 2.055: 0.9294 + 0.287*(ln(FRD) − 2.055) |
|  | Incidence | Log-Anchored Model | 1.85 | 3.03 | 0.16 | 1.85 | 41.44 | Poor fit | E[y] = (1 + FRD)^0.19328 − 0.00346 |
|  |  | Piecewise model (discontinuous GLM) | 1.30 | 2.32 | 0.72 | 1.41 | 21.38 | Poor fit | ln(FRD) ≤ 2.055: -4.2777 + 0.2066*ln(FRD)  //  ln(FRD) > 2.055: -1.5902 + 1.3909*(ln(FRD) − 2.055) |
|  | Bites | Log-Anchored Model | 0.90 | 1.07 | 0.05 | 0.96 | 51.91 | Poor fit | E[y] = (1 + FRD)^0.07215 − 0.056 |
|  |  | Piecewise model (discontinuous GLM) | 0.68 | 0.92 | 0.49 | 0.83 | 42.76 | Poor fit | ln(FRD) ≤ 4.072: -0.7826 + 0.3437*ln(FRD)  //  ln(FRD) > 4.072: -7.7647 + 13.5876*(ln(FRD) − 4.072) |

*RMSE — Root Mean Squared Error, MAE — Mean Absolute Error, RRMSE — Relative Root Mean Squared Error, RRMSE <0.15 were considered “excellent fit”, 0.16 – 0.30 “good fit”, 0.31 – 0.50 “moderate fit”, and >0.5 “poor fit”. Re models use a Gaussian (identity) error family. Rabies-incidence and dog-bite models use Gamma (log-link) families.*

**Table S3: Estimated FRD density (95 % CI) where Re = 1.** Analysis used case detection rate (CDR) scenarios (1 %, 5 %, 10 %) adjusted for the full free-roaming dog population and subset of dogs susceptible to rabies infection for sensitivity analysis.

| **Type** | Model | **FRD at Re = 1 (95% CI)** |
| --- | --- | --- |
| CDR 1% (All) | Log-Anchored Model | 19.8 (16.2-23.3) |
| CDR 1% (All) | Piecewise model (discontinuous GLM) | 12.3 (11.4-17.1) |
| CDR 10% (All) | Log-Anchored Model | 19.8 (16.2-23.9) |
| CDR 10% (All) | Piecewise model (discontinuous GLM) | 12.3 (11.4-17.1) |
| CDR 5% (All) | Log-Anchored Model | 19.3 (15.9-23.3) |
| CDR 5% (All) | Piecewise model (discontinuous GLM) | 12.2 (11.3-16.3) |
| CDR 1% (Susceptible) | Log-Anchored Model | 12.9 (15.9-10.8) |
| CDR 1% (Susceptible) | Piecewise model (discontinuous GLM) | 10.9 (10-13.9) |
| CDR 10% (Susceptible) | Log-Anchored Model | 19.8 (16.2-23.9) |
| CDR 10% (Susceptible) | Piecewise model (discontinuous GLM) | 12.3 (11.4-17.1) |
| CDR 5% (Susceptible) | Log-Anchored Model | 13.7 (11.4-16.8) |
| CDR 5% (Susceptible) | Piecewise model (discontinuous GLM) | 10.8 (10.1-13.9) |
